# Serum Soluble CD40 Ligand in Predicting Simple Appendicitis and Complicated Appendicitis at Different Time Points in Children

**DOI:** 10.3389/fped.2021.676370

**Published:** 2021-06-09

**Authors:** Wun-Yan Huang, Chun-Yu Chen, Yu-Jun Chang, En-Pei Lee, Han-Ping Wu

**Affiliations:** ^1^Department of Pediatric Emergency Medicine, Children Hospital, China Medical University, Taichung, Taiwan; ^2^Department of Medicine, School of Medicine, China Medical University, Taichung, Taiwan; ^3^Laboratory of Epidemiology and Biostastics, Changhua Christian Hospital, Changhua, Taiwan; ^4^Division of Pediatric Critical Care Medicine, Department of Pediatrics, Linko Chang Gung Memorial Hospital, Taoyuan, Taiwan; ^5^College of Medicine, Chang Gung University, Taoyuan, Taiwan; ^6^Department of Medical Research, Children's Hospital, China Medical University, Taichung, Taiwan

**Keywords:** soluble CD40 ligand, appendicitis, children, simple appendicitis, gangrenous appendicitis

## Abstract

**Objectives:** Appendicitis is a common abdominal emergency in children. It is difficult for clinicians to distinguish between simple appendicitis (SA), gangrenous appendicitis (GA), and ruptured appendicitis (RA) in children based on physical and current laboratory tests. Abdominal computed tomography with the disadvantage of excess radiation exposure is usually used in the emergency room for appendicitis surveys. Serum soluble CD40 ligand (sCD40L) is an inflammatory biomarker. This study aimed to use sCD40L to distinguish SA, GA, and RA.

**Methods:** All patients aged <18 years old with suspected appendicitis were tested once for serum sCD40L within 72 h of appendicitis symptoms. We compared sCD40L levels of SA, GA, and RA individually on days 1, 2, and 3 in patients with normal appendix (NA), a total of nine subgroups. Thereafter, the diagnostic performance of sCD40L in predicting appendicitis and the receiver operating characteristic curves were carried out.

**Results:** Of 116 patients, 42 patients had SA, 20 GA, 44 RA, and 10 NA. We found six subgroups with significant *p*-values of sCD40L predicting appendicitis as follows: SA on day 2, GA on days 2 and 3, and RA on days 1–3. The sensitivity and specificity of sCD40L at the best cutoff point with 178 pg/mL in these six subgroups range from 0.75 to 1.00 and 0.90, respectively.

**Conclusions:** SCD40L is a good predictor of pediatric appendicitis. Clinicians can use sCD40L to distinguish from SA, GA, and RA in children with suspected appendicitis.

## Introduction

Appendicitis is one of the most common abdominal emergencies in pediatric patients (1, 2). Its typical symptoms usually shift from periumbilical pain initially to right lower quadrant with fever, nausea, vomiting, anorexia, and a wide variety of atypical symptoms resulting in diagnostic difficulty (3). From mild to severe, appendicitis can be classified as simple appendicitis (SA) and complicated appendicitis including gangrenous appendicitis (GA) and ruptured appendicitis (RA) (4, 5). SA is defined as non-perforated appendicitis without necrotic tissue. GA is an inflamed appendix with grossly necrotic tissue without perforation or abscess. RA is also called perforated appendicitis (6). Early diagnosis and treatment of SA, including antibiotics and operation, can avoid the occurrence of GA and RA. Delayed treatment may lead to many complications including severe peritonitis and septic shock (7).

Due to difficulties in diagnosing atypical appendicitis, many literatures have proposed various diagnostic methods including various serum laboratory tests, scoring system, and imaging. Some research reported that white blood cell (WBC), C-reactive protein (CRP), and urinalysis appearances can predict pediatric appendicitis (7–9). The Pediatric Appendicitis Score and Alvarado score are often used to evaluate the possibility of pediatric appendicitis (10). With the development of imaging, abdominal ultrasound and computed tomography (CT) have become the commonly used tools to diagnose appendicitis (11).

In recent years, many literatures point out the application of a possible biomarker in diagnosing pediatric appendicitis. One research mentioned that IP-10, MIP-1α, and IL-10 showed potential in assessing appendicitis (12). The CD40 ligand (CD40L) and its soluble CD40 ligand (sCD40L) are proteins with prothrombogenic and proinflammatory properties. sCD40L has been regarded as an inflammatory biomarker for sepsis. CD40L belongs to the tumor necrosis factor family. In an inflammatory response, sCD40L induces platelet aggregation and plays an important role in the series of messages during inflammation. The sCD40L level is positively correlated with the severity of the inflammatory response (13). This study aimed to use serum sCD40L to distinguish between SA, GA, and RA in children.

## Materials and Methods

### Patient Population

This was a prospective study that surveyed children with suspected acute appendicitis admitted to the medical center in central Taiwan from August 2012 to July 2015. Pediatric patients aged <18 years old with appendicitis-like symptoms and signs such as abdominal pain, loss of appetite, nausea, vomiting, fever, rebounded tenderness in the right lower quadrant (RLQ) of the abdomen tenderness, and migration of pain were further analyzed. Patients under 4 years of age were excluded. Also, patients whose symptoms and signs lasted more than 3 days were excluded from the analysis. Appendicitis was diagnosed by the faculty physician treating the patients. Patients with suspected appendicitis treated through non-surgical methods were excluded. Histopathologic evaluation was used to confirm appendicitis diagnosis. The study protocol was approved by the Institutional Review Board and ethics committee of Chang Gung Memorial Hospital and was conducted in accordance with the approved guidelines and the Declaration of Helsinki. Informed consent was obtained from a parent and/or legal guardian of children participating in the study. All methods were performed in accordance with relevant protocols.

On admission, all the following parameters were recorded: age, gender, body temperature, time of the onset of symptoms and signs, time of admission, and blood laboratory tests. Every selected patient tested once for serum sCD40L within 72 h of appendicitis symptoms. The duration of patients' symptoms was defined as number of days from the onset of illness to admission to the hospital. The durations were designated as follows: <24 h as day 1, 24 to 48 h as day 2, and 48 to 72 h as day 3. All serum sCD40L levels were measured using an enzyme-linked immunosorbent assay (R&D system, MN, USA). The ultimate diagnosis of patients who were treated through operative methods was based on histologic examination of the excised appendix. According to the histologic findings, patients with a final diagnosis of SA, GA, and RA were selected, and the above parameters were collected for further analysis. Patients excluded from the diagnosis of appendicitis are classified as normal appendices (NAs). Finally, we compared the serum sCD40L levels among patients with NA, SA, GA, and RA. SA was defined as neutrophilic infiltration of mucosa, submucosa and muscularis propria; GA was defined as transmural inflammation with necrosis and mucosal ulceration; and RA was defined as extensive transmural inflammation with perforation. NA was also defended as negative appendectomy. All patients were regrouped into three groups as Group I, Group II, and Group III. Group I aimed to compare levels of sCD40L between NA and SA, Group II aimed to compare levels of sCD40L between GA and NA, and Group III aimed to compare levels of sCD40L between RA and NA.

### Measurement of sCD40L Levels

Plasma sCD40L levels were measured by using specific immunoassays (R & D Systems) ac-cording to the instructions of themanufacturer. In addition, sCD40L concentrations were measured with an sCD40L assay (Quantikine® R&D Systems). According to the package insert, the R&D ELISA was used to measure sCD40L, and was linear within the analytical range of the assay.

### Statistics

The methods of statistical analysis used were Mann-Whitney *U*-test, chi-square test, univariate logistic regression analysis, and receiver operating characteristic (ROC) curves. In the descriptive analysis, values were presented as mean ± standard deviation (SD). The difference between groups was presented as 95% confidence intervals. In univariate logistic regression analysis, odds ratios of sCD40L levels in all appendicitis subgroups compared with the NA group were calculated. An odds ratio estimates = 1 indicated that both the appendicitis group and the NA group had the same odds. Odds ratio estimates >1 may indicate how much higher the odds of sCD40L levels in the appendicitis groups than that in the NA group in developing the outcome over a specified time period. In ROC curves, the test characteristics of these different cutoff values, including sensitivity, specificity, positive likelihood ratio (LR^+^), negative likelihood ratio (LR^−^), and area under the ROC curve (AUC) were also examined. The AUC, calculated using the trapezoidal rule, was considered a global measure of the diagnostic value of the parameter. The best cutoff values could be determined based on the Youden's index (sensitivity + specificity −1). An optimal test result gives a value of 1.0, and a useless test result gives a value of 0.5. LR^+^ and LR^−^ were calculated for the best cutoff values. We used likelihood ratios for analysis instead of positive/negative predictive values because positive/negative predictive values may be affected by the prevalence. Statistical significance was defined at the *p* < 0.05 level, and all statistical analyses were conducted using IBM SPSS Statistics software (version 22.0; SPSS Inc., Chicago, IL, USA).

## Results

During the study period, total of 116 pediatric patients with suspected appendicitis were collated; they comprised 54 boys (46.6%) and 62 girls (53.4%) with a mean age of 11.1 ± 4.2 years. Symptoms and signs which patient first felt ill commonly included migration of abdominal pain, anorexia, nausea and RLQ tenderness in cases with SA and GA, but fever, RLQ tenderness, nausea/vomiting, and rebounded pain over RLQ were common in cases with RA. Of the 116 cases, 42 had histologically proven SA, 20 had GA, 44 had RA, and 10 had NA. All enrolled patients received appendectomy. The final diagnoses of the patients with normal appendices included infectious enteritis, functional gastrointestinal disorders, diverticulitis, intestinal perforation, mesenteric lymphadenopathy, and ruptured tubo-ovarian abscess.

The detection time of serum sCD40L in all patients was recorded. The mean and SD of sCD40L in each group from days 1 to 3 are also listed in Table 1. The mean sCD40L levels in patients with GA and RA increased from days 1 to 3. The highest mean sCD40L level was found in patients with RA on day 3 as 453.06 ± 226.99 pg/mL. However, in patients with SA, the mean sCD40L level on day 2 (264.81 ± 78.64 pg/mL) was higher than that on days 1 and 3 (160.93 ± 48.82 pg/mL; 193.17 ± 88.50 pg/mL, respectively). In Group I, the mean sCD40L levels showed a significant difference on day 2 (*p* < 0.05); in Group II, the mean sCD40L levels were significantly different on days 2 and 3 (both *p* < 0.05); in Group III, the mean sCD40L levels were all significantly different on days 1–3 (all *p* < 0.05).

**Table 1:** Comparison of serum sCD40L among patients with normal appendices, simple appendicitis, gangrenous appendicitis, and ruptured appendicitis on the first 3 days after onset of symptoms.

**Parameter**	**Normal appendices**	**Simple appendicitis**	***P-*value**
	**Mean ± SD (no.)**	**Mean ± SD (no.)**	
**sCD40L (pg/mL)**			
Day 1	145.69 ± 65.41 (10)	160.93 ± 48.82 (22)	0.583
Day 2	145.69 ± 65.41 (10)	264.81 ± 78.64 (12)	0.004
Day 3	145.69 ± 65.41 (10)	193.17 ± 88.50 (8)	0.266
	**Normal appendices**	**Gangrenous appendicitis**	
Day 1	145.69 ± 65.41 (10)	181.95 ± 20.62 (5)	0.086
Day 2	145.69 ± 65.41 (10)	231.97 ± 45.15 (9)	0.007
Day 3	145.69 ± 65.41 (10)	266.71 ± 42.06 (6)	0.010
	**Normal appendices**	**Ruptured appendicitis**	
Day 1	145.69 ± 65.41 (10)	298.96 ± 91.91 (7)	0.001
Day 2	145.69 ± 65.41 (10)	373.48 ± 109.49 (17)	<0.0001
Day 3	145.69 ± 65.41 (10)	453.06 ± 226.99 (20)	<0.0001

**Figure 1:**
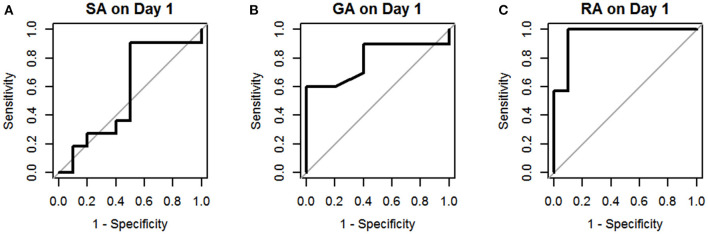
Receiver operating characteristic curves for sCD40L in distinguishing children with acute appendicitis from NA on day 1: SA **(A)**, GA **(B)**, and RA **(C)**.

**Figure 2:**
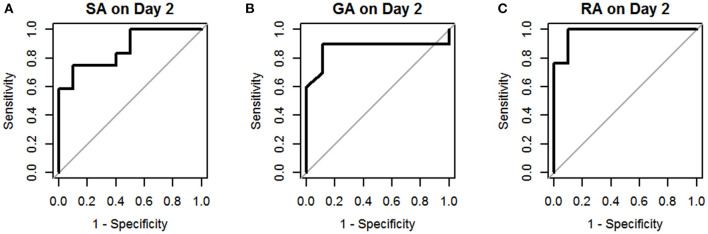
Receiver operating characteristic curves for sCD40L in distinguishing children with acute appendicitis from NA on day 2: SA **(A)**, GA **(B)**, and RA **(C)**.

**Figure 3:**
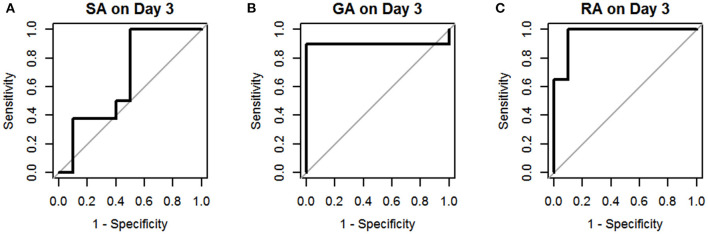
Receiver operating characteristic curves for sCD40L in distinguishing children with acute appendicitis from NA on day 3: SA **(A)**, GA **(B)**, and RA **(C)**.

The results of univariate logistic regression analysis of sCD40L in the subgroups on days 1–3 are shown in Table 2. Nine subgroups of appendicitis were individually compared with the NA group, and we found sCD40L was a significant predictor for SA om day 2, GA on days 2–3, and RA on days 1–3. on (all *p* < 0.05). The findings also showed that in Group I, odds ratio = 1.019 on day 2 may indicate that a one unit increase in sCD40L levels lead to a 1.9% increase in the odds of SA on day 2. The ROC curves are drawn to discriminate acute appendicitis from other acute abdominal diseases on days 1 (Figure 1), 2 (Figure 2), and 3 (Figure 3). The AUCs of ROCs of the above selected six subgroups are all exceed 0.8, showing good discrimination in judgement of appendicitis. Table 3 summarizes their two cutoff values for the discriminators to “rule in” or “rule out” three different stages of appendicitis to confirm or exclude status of appendicitis, such as SA, GA, and RA. In Group I on day 2, the cutoff value for sCD40L of 100.00 pg/mL had the highest sensitivity of 1.00 to rule out SA, while 301.00 pg/mL had the highest specificity of 1.00 to rule in SA. In Group II on days 2 and 3, the cutoff values of sCD40L of 160.00 and 178.00 pg/mL, respectively, had the highest sensitivity to rule out GA, and 301.00 pg/mL had the highest specificity to rule in GA. In Group III on days 1–3, the cutoff values for sCD40L of 178.00 pg/mL had the highest sensitivity of 1.00 to rule out RA, while 301.00 pg/mL had the highest specificity of 1.00 to rule in RA. In addition, we further determined the best cutoff values of sCD40L to predict SA on day 2, GA on days 2–3, and GA on days 1 to 3, as shown in Table 4.

**Table 2:** Logistic regression analysis of sCD40L for the diagnosis of acute appendicitis in the subgroups on days 1–3.

	**Odds ratio**	**95% CI**	***P-*value**
**Group I**			
Day 1	1.006	0.990–1.022	0.458
Day 2	1.019	1.005–1.033	0.009
Day 3	1.009	0.995–1.022	0.205
**Group II**			
Day 1	1.013	0.991–1.036	0.260
Day 2	1.026	1.003–1.048	0.024
Day 3	1.030	1.004–1.056	0.024
**Group III**			
Day 1	1.023	1.003–1.043	0.024
Day 2	1.028	1.006–1.051	0.013
Day 3	1.033	1.005–1.062	0.022

*Group I: Comparison of patients with simple appendicitis and normal appendices.*

*Group II: Comparison of patients with gangrenous appendicitis and normal appendices.*

*Group III: Comparison of patients with ruptured appendicitis and normal appendices*.

**Table 3:** Sensitivity, specificity, LR^+^, and LR^−^ at the two cutoff values of sCD40L to rule out and rule in simple appendicitis, gangrenous appendicitis, and ruptured appendicitis in children.

	**Cutoff values (pg/mL)**	**Sensitivity**	**Specificity**	**LR^**+**^**	**LR^**−**^**	**AUC (95% CI)**
**Group I**						
Day 1	>90.04	0.96	0	0.96	–	0.56 (0.31–0.82)
	>301.00	0	1.00	–	1	
Day 2	>100.00	1.00	0.50	2.00	0	0.87 (0.66–0.97)
	>301.00	0.58	1.00	–	0.42	
Day 3	>100.00	1.00	0.50	2.00	0	0.66 (0.40–0.93)
	>301.00	0	1.00	–	1	
**Group II**						
Day 1	>160.00	1.00	0.6	2.50	0	0.79 (0.58–1.00)
	>301.00	0	1.00	–	1	
Day 2	>160.00	1.00	0.6	2.50	0	0.87 (0.64–0.98)
	>301.00	0	1.00	–	1.00	
Day 3	>178.00	1.00	0.90	10.00	0	0.90 (0.65–0.99)
	>301.00	0	1.00	–	1.00	
**Group III**						
Day 1	>178.00	1.00	0.90	10.00	0	0.96 (0.74–1.00)
	>301.00	0.57	1.00	–	0.43	
Day 2	>178.00	1.00	0.90	10.00	0	0.98 (0.83–1.00)
	>301.00	0.76	1.00	–	0.24	
Day 3	>178.00	1.00	0.90	10.00	0	0.97 (0.83–1.00)
	>301.00	0.65	1.00	–	0.35	

*LR, likelihood ratio.*

*Group I: Comparison of patients with simple appendicitis and normal appendices.*

*Group II: Comparison of patients with gangrenous appendicitis and normal appendices.*

*Group III: Comparison of patients with ruptured appendicitis and normal appendices*.

**Table 4:** Sensitivity, specificity, LR^+^, and LR^−^ at the best cut-off values of sCD40L in distinguishing simple appendicitis, gangrenous appendicitis and ruptured appendicitis from normal appendices in children.

	**Cut-off values (pg/ml)**	**Sensitivity**	**Specificity**	**LR^**+**^**	**LR^**−**^**	**AUC (95% CI)**
**Group I**						
Day 2	>160.00	0.833	0.60	2.08	0.27	0.87 (0.66–0.97)
**Group II**						
Day 2	>168.00	0.89	0.60	4.44	0.14	0.87 (0.64–0.98)
Day 3	>178.00	1.00	0.90	10.00	0	0.90 (0.65–0.99)
**Group III**						
Day 1	>178.00	1.00	0.90	10.00	0	0.96 (0.74–1.00)
Day 2	>178.00	1.00	0.90	10.00	0	0.98 (0.83–1.00)
Day 3	>178.00	1.00	0.90	10.00	0	0.97 (0.83–1.00)

*Group I: Comparison of the patients with simple appendicitis and the patients with normal appendices.*

*Group II: Comparison of the patients with gangrenous appendicitis and the patients with normal appendices.*

*Group III: Comparison of the patients with ruptured appendicitis and the patients with normal appendices*.

## Discussion

The causes of pediatric abdominal pain range from relatively benign etiologies to potentially life-threatening issues. Distinguishing acute abdominal pain from other abdominal diseases is necessary, and appendicitis is one of the most common acute abdominal pain in children. However, distinguishing between acute appendicitis and other abdominal emergencies is particularly difficult for young children (1, 14). Early diagnosis of pediatric appendicitis will not only prevent perforation, abscess formation, and postoperative complications, but may also decrease medical cost by shortening hospitalizations (14, 15). Abdominal CT is the most common tool for diagnosing appendicitis in the emergency department. However, high radiation is a disadvantage of abdominal CT, as it tends to increase the risk of malignancy in children (16). Concerns led to finding new clinical laboratory tests to diagnose pediatric appendicitis. Therefore, this study uses sCD40L as a predictive factor for pediatric appendicitis.

Our study demonstrated the mean and SD of sCD40L in different stages of acute appendicitis such as SA, GA, and RA on days 1–3 for a detailed analysis. Previous research reported that antibiotics as initial treatment for pediatric patients with uncomplicated appendicitis are effective (17). Our study showed that levels of sCD40L may play a crucial role in predicting the severity of appendicitis. This information may then aid in the decision-making to tailor the management option to either surgical or non-surgical accordingly. Many pediatric research studies have reported on different serum biomarkers but with variable diagnostic accuracy in predicting appendicitis. Several studies have reported that sensitivities and specificities of WBC count in predicting pediatric appendicitis vary widely between 0.70–0.80 and 0.60–0.68, respectively (18–21). Some studies reported sensitivities and specificities of CRP level in predicting pediatric appendicitis, ranging from 0.50–0.95 to 0.25–0.85, respectively (21–23). Compared to the above studies, our study showed that sCD40L had favorable sensitivity and specificity in predicting pediatric appendicitis. We believe that sCD40L may serve as a good predictor for acute appendicitis in children.

In addition, we further analyzed predictive values of sCD40L in predicting different stages of acute appendicitis at different time points after onset of symptoms and signs. Based on the results of logistic regression analysis, sCD40L was significantly different in children with SA on day 2 (AUC, 0.87), GA on days 2 and 3 (AUC, 0.87 and 0.90.), and RA on days 1–3 (AUC, 0.96, 0.98, and 0.97, respectively). According to the ROC analysis, the AUCs of the six subgroups were all favorable. In this study, we defined two cutoff points for each significant variable that are easily applicable to answer at what point the variables can rule in and rule out acute appendicitis. Thus, sCD40L predicting GA showed excellent discrimination on each day. In GA on days 2 and 3, sCD40L below 160.00 and 178.00 pg/mL, respectively, can exclude the possibility of GA. Actually, we found that sCD40L at a cutoff value of 160.00 pg/mL is also the best discriminator with 1.00 sensitivity in predicting GA on day 1. We conclude that sCD40L below 160 pg/mL can exclude the possibility of GA from days 1 to 3. Clinically, sCD40L over 301 pg/mL can be considered highly suspected of GA or early stage of complicated appendicitis. Clinicians should be aware of the need to actively treat appendicitis at this point to avoid subsequent complications. Some pediatric studies also reported different biomarkers in predicting complicated appendicitis, and their sensitivities and specificities vary widely between 0.69–0.97 and 0.64–0.83, respectively (24–26). Our study showed that sCD40L had excellent sensitivity and specificity in predicting complicated appendicitis in children. In RA on days 1–3, sCD40L below 178.00 pg/mL can rule out the possibility of RA, which means ruling out perforated appendicitis. In contrast, sCD40L above 301.00 pg/mL can confirm appendicitis and may have a high possibility of RA. In these situations, there may not be a need for further investigation in patients with clinically suspected appendicitis to confirm the diagnosis.

In contrast, it is important to pay more attention to an indeterminate zone in clinical practice because it is not easy for them to make decisions about patients with suspected appendicitis. Once the cutoff point of sCD40L is >160 pg/mL on day 2, the probability of SA will increase. Moreover, once CD40L is >168 pg/mL on day 2 and >178 pg/mL on day 3, the probability of GA will highly increase. Furthermore, once sCD40L is at 178.00 pg/mL cutoff value, its sensitivity and specificity in predicting RA on days 1–3 are all 1.00 and 0.90, respectively. The large AUCs indicate the strong diagnostic capacity for RA.

To our knowledge, this is the first study in which sCD40L has been evaluated as a potential biomarker for predicting pediatric appendicitis. This diagnostic performance of sCD40L in our study was good and may be applied clinically to determine appendicitis in children, especially for complicated appendicitis. In cost effectiveness, the cost of using sCD40L in clinical decision-making for complicated appendicitis may reduce the cost of performing other laboratory tests and more imaging examinations such as CT and abdominal sonography. The mechanism of sCD40L has not been studied clearly in appendicitis. There may be similarities to the mechanisms observed in inflammatory diseases such as sepsis (13, 27). The mechanism of sCD40L in sepsis is as follows: The type I transmembrane receptor protein belongs to the Tumor Necrosis Factor (TNF) receptor superfamily. CD40L is expressed in many cells, including platelets, immune cells, endothelial cells, fibroblasts, and smooth muscle cells. CD40 becomes actively internalized in the cell after binding to CD40L. CD40L will turn into sCD40L in plasma, and sCD40L stored in unstimulated platelets will induce platelet aggregation in a series of inflammatory responses. Then, the nuclear factor-κB (NF-κB) signaling pathway is stimulated with subsequent upregulation of proinflammatory and prothrombotic factors. Thus, sCD40L plays an important role in immune response and inflammation during sepsis (28–31). However, the association between sCD40L and appendicitis needs to be studied further because of its unclear mechanism.

In this study, the main findings showed the diagnostic accuracy of a novel biomarker, sCD40L, in distinguishing children with simple, gangrenous and ruptured appendicitis. The findings suggest sCD40L levels were elevated in patients with appendicitis vs. non-appendicitis. In addition, sCD40L levels increased correspondingly with increased severity of the appendicitis, which the levels could be higher in ruptured appendicitis than simple appendicitis. This may also aid clinicians in the decision-making to tailor the management option to either surgical or non-surgical accordingly.

## Strength and Limitation

Our study had two strengths. First, our study is the first prospective study evaluating diagnostic performance of sCD40L in predicting pediatric appendicitis. Second, our study showed good correlation of sCD40L levels with appendicitis, especially GA and RA. However, there are two limitations in our study. First, the study was performed in a small sample sizes from single hospital. Second, levels of sCD40L were sampled at only one-time point over a 3-days period for each patient. It is not ethical to prolong sampling period, causing delay treatment.

## Conclusion

Serum sCD40L may be useful in predicting acute appendicitis in children, especially for gangrenous and perforated appendicitis. Serum sCD40L has the best discrimination at 178.00 pg/mL in predicting pediatric perforated appendicitis. Serum sCD40L levels below 100.00 pg/mL may exclude appendicitis, and those above 301.00 pg/mL strongly suggest RA in children.

## Data Availability Statement

The original contributions presented in the study are included in the article/supplementary material, further inquiries can be directed to the corresponding author/s.

## Author Contributions

W-YH, E-PL, and H-PW conceived and designed the study. C-YC collected and analyzed the data. Y-JC performed the statistical analysis. W-YH drafted the manuscript. E-PL and H-PW designed and oversaw the study, interpreted the data, and revised the manuscript. All authors have read and approved the final manuscript for publication.

## Conflict of Interest

The authors declare that the research was conducted in the absence of any commercial or financial relationships that could be construed as a potential conflict of interest.

## Abbreviations

AUC: area under the ROC curve
CD40L: CD40 ligand
CI: confidence interval
CRP: C-reactive protein
CT: computed tomography
GA: gangrenous appendicitis
LR: likelihood ratio
NA: normal appendix
NF-κB: nuclear factor-κB
RA: ruptured appendicitis
ROC: receiver operating characteristic
SA: simple appendicitis
sCD40L: soluble CD40 ligand
SD: standard deviation
WBC: white blood cell.
